# Common Variants of Inflammatory Cytokine Genes Are Associated with Risk of Nephropathy in Type 2 Diabetes among Asian Indians

**DOI:** 10.1371/journal.pone.0005168

**Published:** 2009-04-09

**Authors:** Tarunveer Singh Ahluwalia, Madhu Khullar, Monica Ahuja, Harbir Singh Kohli, Anil Bhansali, Viswanathan Mohan, Radha Venkatesan, Taranjit Singh Rai, Kamal Sud, Pawan K. Singal

**Affiliations:** 1 Department of Nephrology, Post Graduate Institute of Medical Education and Research, Chandigarh, India; 2 Department of Endocrinology, Post Graduate Institute of Medical Education and Research, Chandigarh, India; 3 Department of Experimental Medicine and Biotechnology, Post Graduate Institute of Medical Education and Research, Chandigarh, India; 4 Madras Diabetes Research Foundation and Dr. Mohan's Diabetes Specialties Centre, Gopalapuram, Chennai, India; 5 Institute of Cardiovascular Sciences, St Boniface General Hospital Research Centre, University of Manitoba, Winnipeg, Manitoba, Canada; Ohio State University Medical Center, United States of America

## Abstract

**Background:**

Inflammatory cytokine genes have been proposed as good candidate genes for conferring susceptibility to diabetic nephropathy. In the present study, we examined the combined effect of multiple alleles of pro inflammatory cytokine genes for determining the risk of nephropathy in type 2 diabetic patients.

**Methodology/Principal Findings:**

Eight single nucleotide polymorphisms (SNPs) of pro-inflammatory cytokine genes (*CCL2*, *TGFB1*, *IL8, CCR5,* and *MMP9*) were genotyped in two independently ascertained type 2 diabetic cohorts with (DN) and without nephropathy (DM); consisting of patients from North India (n = 495) and South India (n = 188). Genotyping was carried out using PCR, allele specific oligonucleotide-PCR (ASO-PCR), PCR-RFLP and TaqMan allelic discrimination assays and the gene–gene interaction among genetic variants were determined by multi dimensional reduction (MDR) software. Serum high sensitive CRP (hs-CRP) levels were measured by ELISA. The hs-CRP levels were significantly higher in DN as compared to the DM group (p<0.05). The *CCL2*, *IL8*, *CCR5* and *MMP9* polymorphisms were found to be associated with the risk of diabetic nephropathy. Frequency of *CCL2* II, *IL8* -251AA, *CCR5* 59029AA and MMP9 279Gln/Gln genotypes were significantly higher in DN than in DM group (p<0.05) and associated with an increased risk of nephropathy in both North and South Indian cohorts. *CCR5* DD and *IL8* -251AA genotypes were more prevalent in North Indian DN group only. The co-occurrence of risk associated genotypes (II, -2518GG (*CCL2*), DD (*CCR5*) and 279Gln/Gln (*MMP9*) conferred a tenfold increased risk of nephropathy among type 2 diabetics (p<0.0002).

**Conclusion:**

The present study highlights that common variants of inflammatory cytokine genes exert a modest effect on risk of DN and a combination of risk alleles confer a substantial increased risk of nephropathy in type 2 diabetes among Asian Indians.

## Introduction

Genetic susceptibility plays an important role in the pathogenesis of diabetic nephropathy (DN) and multiple genetic approaches, including candidate gene association studies and the genome-wide association study (GWAS) are being pursued to identify the susceptibility gene(s) for DN [1]–[7]. In recent years, it has become evident that inflammatory mechanisms contribute significantly to the development and progression of DN. These include the infiltration of renal compartments by lymphocytes and monocytes/macrophages as well as local production of cytokines and chemokines in the kidney [8], [9]. Although the mechanisms underlying the regulation of these cytokines in the kidneys of patients with diabetes mellitus remain unclear, it has been proposed that genetic variations in the genes encoding the inflammatory cytokines might confer susceptibility to DN [10] by altering their functions or expressions. For example, a promoter polymorphism (-2518A/G) in the *CCL2* gene was found to be associated with progressive kidney failure in Koreans with type 2 diabetes [11] and *TGFB1* T869C gene polymorphism showed association with nephropathy in type 2 diabetic Chinese patients [12]. Polymorphisms in the *CCR5* and *MMP9* have been also reported to be associated with the increased risk of nephropathy [5], [13]–[15]. In a polygenic complex disorder like DN, association of individual polymorphisms in genes may be small and sometimes non-informative, whereas specific combinations of specific genotypes may be more relevant. However, few studies have examined the multiple alleles simultaneously for determining the risk of DN [16], [17]. In the present study, we investigated combinatorial polymorphisms among five inflammatory genes (*TGFB1:* T869C (Leu10Pro) and Tyr81His; *CCL2*: A-2518G and Insertion/Deletion (I/D); *CCR5*: Insertion/Deletion (I/D) and G59029A; *IL8*: T-251A; *MMP9*: Arg279Gln (G>A)) for association with the risk of susceptibility to diabetic nephropathy.

## Results

Demographic and clinical characteristics of the subjects enrolled in the study are given in Table 1. The mean duration of type 2 diabetes (considered with respect to date of detection of diabetes) was similar in diabetic nephropathy and diabetes without nephropathy group (DM) (16 vs.15 years). Percent frequency of retinopathy was higher in DN (76.2%) as compared to DM (37.7%) group.

**Table 1:** Demographic profile of type 2 diabetes subjects (with & without) nephropathy among North India and South India.

North Indian Cohorts			
	Type 2 diabetes subjects		a vs b
	With nephropathy^a^ (n = 240)	Without nephropathy^b^ (n = 255)	
**Age (years)**	60.12±6.2	58.1±8.0	0.32^d^
**Males/Females (%)**	94/146 (39/61)	105/150 (41/59)	1.0^e^
**Mean duration of diabetes (years)**	16.3±3.3	15.62±5.25	>0.05^d^
**Mean arterial S.B.P. (mmHg)** *	145.4±22.5	135.08±17.7	<0.001^c^
**Mean arterial D.B.P. (mmHg)** *	88.8±10.2	84.21±7.51	<0.001^c^
**BMI (Kg/m^2^)** *	27.8±2.9	23.95±2.95	<0.001^c^
**hs-CRP (mg/L)**	2.65±1.2	1.89±1.1	<0.01^d^
**S. Creatinine (mg%)** *	4.22±2.07	1.14±0.11	<0.001^d^
**Blood Urea (mg%)** *	106.7±46.2	36.2±15.2	0.00^d^
**Random blood sugar (mg/dl)**	182.7±53.5	181.3±56.5	0.78^d^
**Total cholesterol (130–250 mg%)**	255.3±64.1	259.24±63.7	0.88^d^
**Tryglycerides (mg%)**	204.8±39.2	207.6±38.2	0.85^d^
**HDL (mg%)**	63.5±15.1	61.05±16.08	0.75^d^
**Retinopathy (%)** *	76.2	37.7	<0.05^e^

South Indian Cohorts			
	With nephropathy^a^ (n = 96)	Without nephropathy^b^ (n = 92)	a vs b
**Age (years)**	60.5±8.7	53.5±12.1	0.08^d^
**Males/Females (%)**	53/43 (55/45)	47/45 (51/49)	0.97^e^
**Mean duration of diabetes (years)**	17.4±7	13.0±2.8	>0.05^d^
**Mean arterial S.B.P. (mmHg)** *	143.5±20.5	127.4±17.6	<0.001^c^
**Mean arterial D.B.P. (mmHg)** *	85.1±10.5	77.2±12.2	<0.001^c^
**BMI (Kg/m^2^)** *	24.5±3.7	25.8±4.0	0.06^c^
**S. Creatinine (mg%)** *	5.5±1.3	1.0±0.2	<0.001^d^
**Blood Urea (mg%)** *	64±18.8	22.5±5.8	<0.001^d^
**Fasting blood sugar (mg/dl)**	173±69	161±59	0.15^d^
**Total cholesterol (130–250 mg%)**	176±74	192±37	0.26^d^
**Tryglycerides (mg%)**	175±88	165±84	0.23^d^
**HDL (mg%)**	43±10	44±10	0.97^d^

* p<0.05; Values are mean±standard deviation.

a Type 2 diabetic with nephropathy.

b Type 2 diabetic without nephropathy.

c Mann-Whitney *U* test.

d Student's *t*-test.

e Pearson's Chi square test.

### High sensitivity- C Reactive Protein (hs-CRP) levels

The mean plasma hs-CRP levels were significantly higher in cases (2.65±1.2 mg/L; p<0.05) as compared to controls (1.89±1.1 mg/L) (Table 1). hs-CRP levels were not associated with any particular genotype within the case or control groups as per logistic regression analysis and *t*-test performed (data not shown).

### Genetic Analysis (North Indian Cohort)

**Figure 1:**
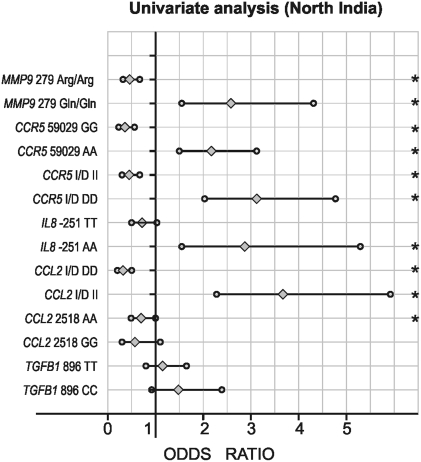
Univariate logistic regression analysis in type 2 diabetic patients with *TGFB1* 869 (T>C), *CCL2* -2518 (A>G), *CCL2* (Del.>Ins.), *IL8* -251 (T>A), *CCR5* Del 32 (Ins.>Del.), and *MMP9* Arg279Gln (G>A) polymorphisms as independent and diabetic nephropathy as a dependent variable among North Indian Cohort. *p<0.05.

**Figure 2:**
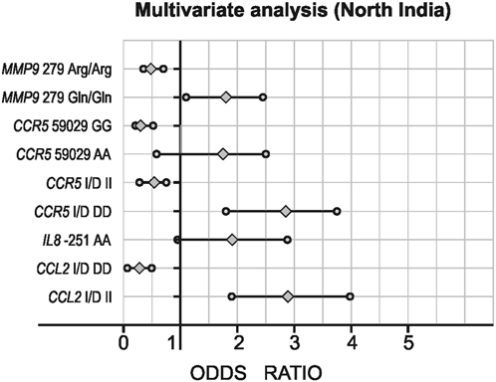
Multivariate logistic regression analysis in type 2 diabetic patients with *TGFB1* 869 (T>C), *CCL2* -2518 (A>G), *CCL2* (Del.>Ins.), *IL8* -251 (T>A), *CCR5* Del 32 (Ins.>Del.), and *MMP9* Arg279Gln (G>A) polymorphisms as independent and diabetic nephropathy as a dependent variable among North Indian Cohort. *p<0.05.

All the polymorphisms examined in this study were in Hardy-Weinberg equilibrium in both set of populations. Allele and genotype frequencies of the polymorphisms of the *TGFB1*, *CCL2*, *IL8*, *CCR5*, and *MMP9* genes are given in Table 2. The genotype frequencies were different between DN and DM groups. Five of the eight variant alleles showed significantly higher allele frequency (*CCL2* Insertion (I): p<0.0001; *IL8* -251A: p<0.007; *CCR5* Deletion (D): p<0.0001, *CCR5* 59029A: p<0.0001; *MMP9* 279Gln: p<0.0001) in DN as compared to DM group. When genotypes were stratified according to dominant and recessive models, after the multiple logistic regression analysis (Figure 1, and 2), five genotypes (Insertion/Insertion (II) of *CCL2*; AA of *IL8*; Deletion/Deletion (DD) and 59029AA of *CCR5*; and AA of *MMP9*) showed significant association with high risk of DN (p<0.05), whereas three genotypes (Deletion/Deletion (DD) of *CCL2*; Insertion/Insertion (II) of *CCR5*; and Arg/Arg of *MMP9*) showed significant association with decreased risk of DN (p<0.05). None of the covariates (age, sex, duration of diabetes, and blood pressure) except blood urea and serum creatinine (p<0.05) were found to be associated with the DN (data not shown).

**Table 2:** Allele and genotype frequencies of the inflammatory gene SNPs in North and South Indians.

North Indians				
Polymorphisms	Allele Frequency		OR(95%CI)	p value
	DM (2n = 510)	DN (2n = 480)		
***TGFB1*** **: rs1800470**	T = 362 (0.71)	T = 331 (0.69)	1.1 (0.83–1.44)	0.48
**869 T>C (Leu10Pro)**	C = 148 (0.29)	C = 149 (0.31)		
**TGFB1**	T = 497 (0.975)	T = 466 (0.97)	1.14 (0.53–2.46)	0.71
**Tyr81His (T>C)**	C = 13 (0.025)	C = 14 (0.03)		
***CCL2*** **: rs1024611**	A = 342 (0.67)	A = 317 (0.66)	1.04 (0.8–1.36)	0.74
**-2518 A>G**	G = 168 (0.33)	G = 163 (0.34)		
***CCL2*** **: rs3917887** *	I = 250 (0.49)	I = 326 (0.68)	2.03 (1.57–2.63)	<0.0001
**Ins./Del. (D>I)**	D = 260 (0.51)	D = 154 (0.32)		
***IL8*** **: rs4073** *	T = 362 (0.71)	T = 302 (0.63)	1.44 (1.1–1.88)	0.006
**-251 T>A**	A = 148 (0.29)	A = 178 (0.37)		
***CCR5*** **: Del 32** *	I = 250 (0.49)	I = 130 (0.27)	2.58 (1.98–3.37)	<0.0001
**Ins./Del.(I>D)**	D = 260 (0.51)	D = 350 (0.73)		
***CCR5*** **: 59029 G>A** *	G = 260 (0.51)	G = 153 (0.32)	2.22 (1.71–2.87)	<0.0001
	A = 250 (0.49)	A = 327(0.68)		
**MMP9: rs17576** *	G = 342 (0.67)	G = 254 (0.53)	1.81 (1.40–2.34)	<0.0001
**(Arg279Gln) G>A**	A = 168 (0.33)	A = 226 (0.47)		

Polymorphisms	Genotype Frequency		p value
	DM (n = 255)	DN (n = 240)	
***TGFB1*** **: rs1800470**	TT = 144 (0.565)	TT = 144 (0.60)	
**869 T>C (Leu10Pro)**	TC* = 74 (0.29)	TC = 41 (0.17)	0.009
	CC = 37 (0.145)	CC = 55 (0.23)	0.1
***TGFB1*** **: Tyr81His (T>C)**	TT = 242 (0.95)	TT = 226(0.94)	
	TC = 13 (0.05)	TC = 14 (0.06)	0.71
	CC = 0 (0)	CC = 0 (0)	-
***CCL2*** **: rs1024611**	AA = 122 (0.48)	AA = 94 (0.39)	
**-2518 A>G**	AG* = 97 (0.38)	AG = 130(0.54)	0.003
	GG = 36 (0.14)	GG = 16 (0.07)	0.09
***CCL2*** **: rs3917887**	DD = 89 (0.35)	DD = 36 (0.15)	
**Ins./Del. (D>I)**	ID* = 82 (0.32)	ID = 79 (0.33)	0.0005
	II* = 84 (0.33)	II = 125 (0.52)	<0.0001
***IL8*** **: rs4073**	TT = 130 (0.51)	TT = 103(0.43)	
**-251 T>A**	TA = 107 (0.42)	TA = 96 (0.40)	0.51
	AA* = 18 (0.07)	AA = 41 (0.17)	0.0005
***CCR5*** **: Del 32**	II = 94 (0.37)	II = 50 (0.21)	
**Ins./Del. (I>D)**	ID = 64 (0.25)	ID = 29 (0.12)	0.57
	DD* = 97 (0.38)	DD = 161(0.67)	<0.0001
***CCR5*** **: 59029 G>A**	GG = 90 (0.35)	GG = 40 (0.16)	
	GA = 80 (0.31)	GA = 75 (0.31)	0.52
	AA* = 85 (0.34)	AA = 125 (0.52)	<0.0001
***MMP9*** **: rs17576**	GG = 122(0.48)	GG = 72(0.30)	
**(Arg 279 Gln) G>A**	GA* = 97 (0.38)	GA = 113(0.47)	0.0007
	AA* = 36 (0.14)	AA = 55 (0.23)	0.0002

South Indians				
Polymorphisms	Allele Frequency		OR(95%CI)	p value
	DM (2n = 184)	DN (2n = 192)		
***CCL2*** **: rs3917887** *	I = 92 (0.5)	I = 121 (0.63)	1.7 (1.12–2.57)	0.01
**Ins./Del. (D>I)**	D = 92 (0.5)	D = 71 (0.37)		
***IL8*** **: rs4073**	T = 134 (0.73)	T = 123 (0.64)	1.5 (0.96–2.33)	0.06
**-251 T>A**	A = 50 (0.27)	A = 69 (0.36)		
***CCR5*** **: Del 32**	I = 151 (0.82)	I = 161 (0.84)	0.88 (0.51–1.5)	0.64
**Ins./Del.(I>D)**	D = 33 (0.18)	D = 31 (0.16)		
***CCR5*** **: 59029 G>A** *	G = 99 (0.54)	G = 67 (0.35)	2.17 (1.43–3.29)	0.0002
	A = 85 (0.46)	A = 125 (0.65)		
***MMP9*** **: rs17576** *	G = 116 (0.63)	G = 84 (0.44)	2.19 (2.45–3.31)	0.0001
**(Arg 279 Gln) G>A**	A = 68 (0.37)	A = 108 (0.56)		

Polymorphisms	Genotype Frequency		p value
	DM (n = 92)	DN (n = 96)	
*CCL2*: rs3917887	DD = 29 (0.32)	DD = 20 (0.21)	
Ins./Del. (D>I)	ID = 34 (0.36)	ID = 32 (0.33)	0.41
	II* = 29 (0.32)	II = 44 (0.46)	0.03
*IL8*: rs4073	TT = 58 (0.63)	TT = 36 (0.38)	
-251 T>A	TA* = 19 (0.21)	TA = 51 (0.53)	<0.0001
	AA = 15 (0.16)	AA = 9 (0.09)	0.92
*CCR5*: Del 32	II = 64 (0.69)	II = 69 (0.72)	
Ins./Del. (I>D)	ID = 23 (0.26)	ID = 23 (0.24)	0.82
	DD = 5 (0.05)	DD = 4 (0.04)	0.73
*CCR5*: 59029 G>A	GG = 35 (0.38)	GG = 20 (0.21)	
	GA = 30 (0.33)	GA = 28 (0.29)	0.26
	AA* = 27 (0.29)	AA = 48 (0.50)	0.003
*MMP9*: rs17576	GG = 42 (0.46)	GG = 28 (0.29)	
(Arg 279 Gln) G>A	GA = 31 (0.34)	GA = 29 (0.30)	0.34
	AA* = 19 (0.20)	AA = 39 (0.41)	0.002

* p<0.05; Genotype frequency calculation is with respect to the homozygous wild type genotypes in each group.

### Genetic analysis (South Indian cohort)

**Figure 3:**
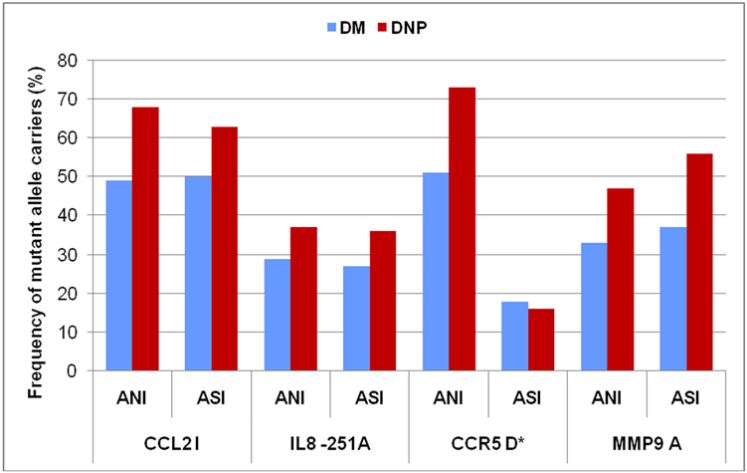
Genotype frequency comparison between Asian type 2 diabetic North Indians (ANI), and South Indians (ASI) with (DN) and without nephropathy (DM) for the four inflammatory variants. *p<0.05 Asian North Indians (ANI) vs. Asian South Indians (ASI). DM: Diabetes without nephropathy; DN: Diabetes with nephropathy.

**Figure 4:**
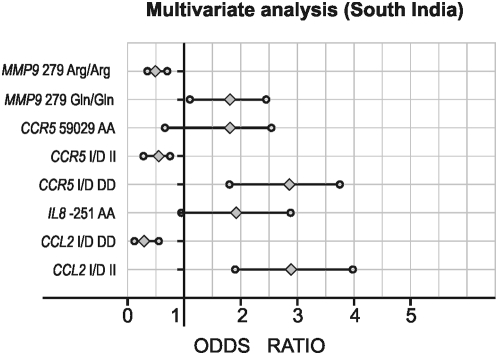
Univariate logistic regression analysis in type 2 diabetic patients with *TGFB1* 869 (T>C), *CCL2* -2518 (A>G), *CCL2* (Del.>Ins.), *IL8* -251 (T>A), *CCR5* Del 32 (Ins.>Del.), and *MMP9* Arg279Gln (G>A) polymorphisms as independent and diabetic nephropathy as a dependent variable among South Indian Cohort. *p<0.05.

**Figure 5:**
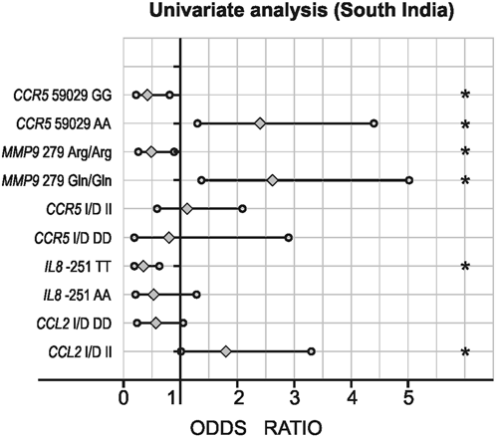
Multivariate logistic regression analysis in type 2 diabetic patients with *TGFB1* 869 (T>C), *CCL2* -2518 (A>G), *CCL2* (Del.>Ins.), *IL8* -251 (T>A), *CCR5* Del 32 (Ins.>Del.), and *MMP9* Arg279Gln (G>A) polymorphisms as independent and diabetic nephropathy as a dependent variable among South Indian Cohort. *p<0.05.

Figure 3 shows the mutant allele frequency comparison between of north Indian and south Indian subjects. The Dominant/Recessive models after logistic regression analysis are shown in figures 4 and 5. No significant differences in either genotype or allele frequencies were observed between the two groups, except for *CCR5* I/D polymorphism; minor allele frequency (MAF) was higher in north Indians as compared to South Indians.

#### Power calculations

Power assuming α = 0.05 and relative risk of 1.4 was 62%, 83%, 85% and 79% for type 2 diabetic patients with diabetic nephropathy for *IL8, CCL2, CCR5, MMP9* polymorphisms (p<0.05) among North Indian population.

### Gene–gene interaction

**Figure 6:**
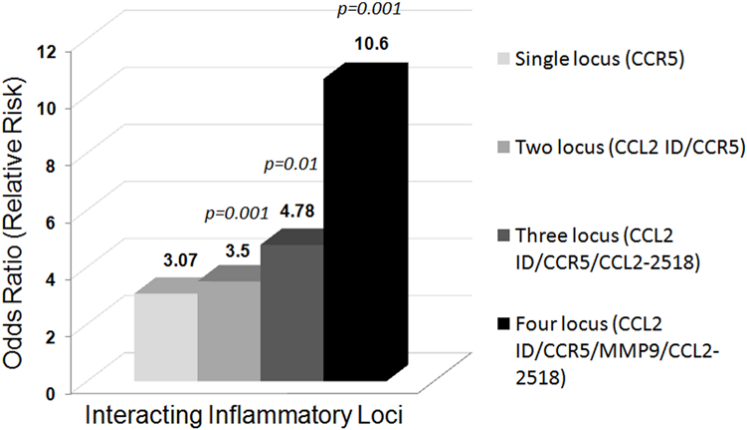
Gene–Gene interaction: Comparison between the number of interacting locus/loci and the odds ratio (relative risk) pertaining to each SNP combination. Testing Accuracy for different combinations: single locus, 63.7 %; two locus, 65.1%; three locus, 70.7%; four locus, 76.4%.

We analyzed the presence of epistatic interactions amongst inflammatory genes, evaluating the possible (one-to-four way) SNP combinations using the multiple dimensionality reduction approach (MDR). All the four models (one, two, three & four locus) had a prediction accuracy of >63% and showed significant interactions (p<0.05), as determined empirically by permutation testing. Four loci SNP combination of -2518AG (*CCL2*), II/ID (*CCL2* I/D), 279Gln/Arg (*MMP9*), and DD (*CCR5* I/D) genotypes was found to be the best model for DN risk prediction (accuracy 0.764; p = 0.0001, Figure 6). The predicted odds ratio of this model was 10.6 (95% CI: 6.6–17.0). The two loci model comprising II genotype (*CCL2*) and DD genotype (*CCR5*) and three loci combination comprising of II (*CCL2* I/D), DD (*CCR5* I/D), and -2518AG (*CCL2*) were also significantly associated with three and five folds risk of DN (p = 0.0001; p<0.02).

## Discussion

Recent studies suggest inflammation to be an essential component of type 2 DM and its complications. We measured hs-CRP as a marker of inflammation in our diabetic cohort and found its levels to be significantly higher in diabetic patients as compared to controls and in nephropathy group as compared to diabetic subjects without nephropathy indicating inflammation to be a relevant factor in the pathogenesis of DN. Our results are consistent with an earlier study which has also reported increased hs-CRP levels in diabetics with proteinuria [18].

Different inflammatory molecules, including pro-inflammatory cytokines have been proposed as critical factors in the development of microvascular diabetic complications, including nephropathy [19]. It has been suggested that genetic variations in the genes encoding the inflammatory cytokines might confer susceptibility to DN by altering the function and/or expression of these cytokines. We investigated the association of genetic polymorphism(s) in inflammatory genes with the risk of diabetic nephropathy and whether co-occurrence of risk conferring variants of inflammatory genes were associated with increased risk of diabetic nephropathy in Asian Indian type 2 diabetic subjects. The key finding of our study was that polymorphisms in *IL8*, *CCL2*, *CCR5*, and *MMP9* genes were associated with increased risk of nephropathy in Asian Indian type 2 diabetics and co-occurrence of specific risk genotypes of these genes conferred several fold greater risk of diabetic nephropathy.

We examined eight variants of five pro-inflammatory cytokine genes which have been either shown to be linked with DN in other ethnic populations or were functional variants affecting expression or function of these genes. All the examined genotypes/alleles were in Hardy-Weinberg Equilibrium in both DN and DM groups. We observed that specific genotypes of all the genes except *TGFB1*, showed independent association with risk of DN.

CCL2, also known as Monocyte chemo-attractant protein-1 (MCP-1), is the strongest known chemo-tactic factor for monocytes and is upregulated in DN. Recent studies suggest that this cytokine is involved in the pathogenesis of diabetic nephropathy [20]. We examined two polymorphisms, *CCL2* A-2518G and *CCL2* Insertion/Deletion in CCL2 gene for association with DN. *CCL2* -2518AG and *CCL2* II genotypes showed two fold higher risk of DN as compared to DM group. The -2518A allele (*CCL2*) was reported to be associated with kidney failure in Korean type 2 DM patients. This SNP has been shown to influence *CCL2* transcriptional activity, with increased CCL2 protein expression in GG/AG genotype carriers as compared to AA carriers in response to IL1B [13], [21]. The *CCL2* I/D polymorphism has been recently reported to be associated with development of inflammatory myopathies [22]. This is the first report of this variant being examined for association with type 2diabetes or nephropathy among Asian Indians.

A common 32-bp deletion mutation (*CCR5* del 32) and a promoter polymorphism CCR5 59029 (G/A) of the CCR5 gene have recently been reported to be associated with the increased risk of nephropathy in type 1 [21] and type 2 diabetic subjects [15]. We too observed a significant association of *CCR5* 59029AA variants with increased risk of DN in our North Indian cohort. Our results are in conformity with other available reports of association of this marker with DN among Asian Indians [6] and Japanese population [14], [15] and suggest that the *CCR5* 59029 A allele may be an independent risk factor for diabetic nephropathy in patients with type 2 diabetes. *CCR5* 59029A-genotype has been shown to be associated with increased *CCR5* expression by peripheral blood mononuclear cells has been seen in individuals with the [23], thereby suggesting that the genotype could regulate *CCR5* gene expression. We found *CCR5* D allele to be associated with increased risk of DN in our North Indian cohort. Limited literature exists regarding the role of this polymorphism in diseases other than AIDS. Male carriers of the 32-bp deletion have been found to be at significantly higher risk of diabetic nephropathy than non-carriers in type 1 diabetes [21]. This deletion alters the open reading frame of the gene resulting in a truncated protein [24]. Thus, a genetically determined impairment in the function of the chemokine receptor CCR5 may change the profile of recruited cells, and this may promote, for example, renal fibrosis instead of normal tissue repair [25].

We found -251AA genotype (*IL8*) to be associated with higher risk of DN. The *IL8* T-251A variant lies in the regulatory region and increases the gene expression [26], suggesting that this genotype could regulate IL8 gene expression. Increased urinary excretion of IL8 has been reported in DN patients [27]. This variant has been associated with inflammatory renal injury [28]. Ours is the first study showing an association of this allele with increased risk of diabetic nephropathy.

The *MMP9* Arg279Gln polymorphism lies in the substrate binding region and decreases binding affinity of type IV collagen to *MMP9* [29]. *MMP9* 279Gln/Gln genotypes was found to be associated with increased risk of DN suggesting that decreased affinity of type IV collagen to MMP9 may lead to decreased degradation leading to over-accumulation of such extracellular matrix (ECM) proteins and contributing to renal damage [30]. However, further studies are required to confirm the functional impact of this variant on diabetic nephropathy.

*TGFB1* Tyr81His polymorphism is a functional SNP in the Exon 2 of *TGFB1* gene and has been previously studied in Asian Indians with chronic renal insufficiency (6). This SNP was found to be monomorphic in our population and was further not analysed. The minor allele frequency (MAF) of the second *TGFB1* SNP T869C (Leu10Pro) was similar in cases and controls (p>0.05). Similar results have been reported in Japanese [31] and Caucasians [32] where, no association between this SNP and DN was observed. However, Wong et al. [33] found 869CC genotype to be associated with DN in Chinese diabetic subjects. The observed heterogeneity in these results could be due to ethnic differences in these studies.

None of the polymorphisms in our study showed any association with hs-CRP levels. A lack of association between IL6 G-174C or IL1 RA genotypes and significant association between CRP and diabetic nephropathy has been reported earlier too, indicating that elevated CRP levels are associated with diabetic nephropathy independent of polymorphisms in cytokine genes [34].

We replicated genotypic associations seen in our cohort in an independently recruited set of diabetic patients from Southern India. We observed that the MAF of the four of risk conferring SNPs (*CCL2* ID, *CCR5* G59029A, *MMP9* Arg279Gln, and *IL8* T-251A) were similar in North and South Indian populations and were associated with increased risk of DN. However, the ‘D’ allele frequency (*CCR5* I/D polymorphism) was higher in North Indian DN group as compared to the South Indian DN group, and did not show association with the risk of nephropathy in the latter. These differences may be because of the ethnic variation between the North and South Indian populations [35], [36] and/or comparatively small sample size of South Indian subjects in our cohort. However, the replication of our data in two independent sets of populations suggests that these associations might be significant.

We analyzed the interaction of the individual genotypes for conferring nephropathy risk using multifactor dimensionality reduction (MDR) software. MDR method was developed by Hahn et al [37] for collapsing high-dimensional genetic data into a single dimension thus permitting interactions to be detected in relatively small sample sizes. It identifies combinations of multilocus genotypes and discrete environmental factors (non-linear interactions) that are associated with a particular disease. It calculates the testing accuracy/Prediction error after taking into account the number of samples. It has been used in several other similar studies [38]. We observed significant epistatic interactions between *CCL2*, *CCR5* and *MMP9* gene SNPs. A four loci combination of genotypes: [-2518AG (*CCL2*)+II/ID (*CCL2* I/D)+279Arg/Gln (*MMP9*)+DD (*CCR5* I/D)] was associated with ten fold increased risk of DN as compared to the risk conferred by individual genotypes, suggesting a synergistic interaction between these genes towards disease susceptibility.

A relatively small sample size such as ours may be a limitation for genetic studies; however, few investigators (mostly multi-centric) have access to larger sample sizes. The strength of our study is that we had an ethnically homogenous and well phenotyped diabetic subjects enrolled from a single centre, thereby reducing phenotyping errors and bias. The inclusion criterion of absence of microalbuminuria on two or more occasions for the control group ensured a good selection of control diabetic group for the study. Also, in accordance with the suggestions of Hattersley and McCarthy [39], the present study fulfils most of the prerequisites for a good genetic association study. Further, most of the variants showing positive association had a minimum power of 76%, which has been shown to be adequate for association studies [40], [41].

In summary, our study provides evidence of the individual risk associated with the inflammatory gene variants and also emphasizes on the combinatorial effect of alleles of the *CCL2* (I allele of I/D polymorphism), *CCR5* (D allele of I/D polymorphism) and *MMP9* (279Gln allele of Arg279Gln) genes for diabetic nephropathy, considering the epistatic effects of individual genes, while investigating susceptibility genes in complex diseases like DN among Asian Indians.

## Materials and Methods

### Subject Selection

Two independently recruited groups of Type 2 diabetes (T2DM) patients and control subjects were evaluated. The first group consisted of unrelated 255 individuals with T2DM and no nephropathy (DM) and 240 consecutive diabetic nephropathy patients (DN), from North India, attending outpatient diabetes and nephrology clinics at Post Graduate Institute of Medical Education and research (PGIMER), Chandigarh, India. Replication studies were performed on a population of 92 cases with T2DM without nephropathy and 96 DN patients recruited from Madras Diabetes Research foundation (MDRF), Chennai, South India enrolled in Chennai Urban Rural Epidemiology Study (CURES) [32]. An informed written consent was taken from all the subjects participating in this study.

Type 2 diabetes was diagnosed on the basis of the WHO criteria. The patients were divided into subgroups: type 2 diabetes patients who had the disease for at least 10 years but remained free of nephropathy (DM) and patients with nephropathy (DN). Diabetic nephropathy status was determined on the basis of a questionnaire, clinical features and laboratory data. Type 2 diabetic subjects diagnosed with either of the following: a) Urinary albumin >300 mg/L or, b) an albumin:creatinine ratio >300 µg/mg without any clinical or laboratory evidence of other kidney disease, formed the diabetic nephropathy group. Type 2 diabetes subjects with duration of onset of 10 years or more and negative for urinary protein (proteinuria) and urinary albumin levels <30 mg/L (measured on two consecutive occasions) formed DM group. It was ensured that the diabetic nephropathy subjects had no microscopic hematuria. In addition, the diabetic subjects (without proteinuria) on anti-hypertensive drug treatment were excluded from the study group in order to avoid misclassification of phenotype. All the subjects in both groups were age, & ethnicity matched. The research carried out was in compliance with the Helsinki Declaration.

### Ethics Statement

The study was approved by the Ethics committee of Post Graduate Institute of Medical Education and Research, Chandigarh, India and also by the Ethics committee of Madras Diabetes Research Foundation, Chennai, India.

### SNP Selection and Genotyping Analysis

The genomic DNA was isolated from peripheral blood, using the Proteinase K-chloroform-phenol method. A total of 8 SNPs of five candidate genes (*TGFB1, CCL2, CCR5, IL8,* and *MMP9*) were selected for the present study, SNPs were chosen based either on their location or functional nature (promoter or exonic) or on their widely analyzed status. Details including location of SNPs in their respective genes, primer sequences, PCR conditions and restriction enzyme with product sizes are given in Table 3. The digested PCR products were run on 12% polyacrylamide gel followed by silver staining.

**Table 3:** Standard PCR conditions used in genotyping the inflammatory gene variants.

SNP (rs#) *Gene*	Primers	Amplicon (bp)	Annealing temp. (°C)/[MgCl_2_] (mmol)	Restriction Enz./Allele size
**869T>C (Leu10Pro) (rs1800470)**	P1: 5′-TTC AAG ACC ACC CAC CTT CT-3′	443	68.2/1.5	Msp A1I (NEB)
***TGFB1***	P2: 5′-TCG CGG GTG CTG TTG TAC A-3′			T = 285, 67, 50, 41 bp
**Exon 1**				C = 273, 67, 50, 41, 12 bp
**Tyr81His (T>C)**	P1: 5′-CCA GAT CCT GTC CAA GCT G-3′	198	57.9/2.5	Rsa I (NEB)
***TGFB1***	P2: 5′-TGG GTT TCC ACC ATT AGC AC-3′			T = 109, 89 bp
**Exon 2**				C = 198 bp
**-2518 A>G**	P1: 5′-TCT CTC ACG CCA GCA CTG ACC-3′	234	59.4/3.0	Pvu II (NEB)
**(rs1024611)**	P2: 5′-GAG TGT TCA CAT AGG CTT CTG-3′			G = 159, 75 bp
***CCL2***				A = 234 bp
**Promoter**				
**Ins./Del.**	P1: 5′-GCT GAT CTT CCC TGG TGC TGA T-3′	202/188	61.3/3.0	I = 202 bp
**(rs3917887)**	P2: 5′- CAT TAA ATC CCA GTG CTT CTG CCT A -3′			D = 188 bp
***CCL2***				
**Intron 1**				
**Del 32**	P1: 5′-GAA GTT CCT CAT TAC ACC TGC AGC TCT C-3′	174/142	63.5/3.5	I = 174 bp
***CCR5***	P2: 5′- CTT CTT CTC ATT TCG ACA CCG AAG CAG AG-3′			D = 142 bp
**Promoter**				
**59029 G>A**	P1: 5′-CAG TCA ACC TGG GCA AAG CC-3′	453	56.5/2.5	Bst 1286I (NEB)
***CCR5***	P2: 5′-AGC TTT GGT CCT GAG AGT CC-3′			G = 408, 45 bp
				A = 453 bp
**Promoter**				
**-251 T>A**	P1: 5′(1A)-CTA GAA ATA AAA AAG CAT ACA A -3′	240/250	53.8/3.0	A = 240 bp
**(rs4073)**	P2: 5′(2T)-ATA AAG TTA TCT AGA AAT AAA AAA GCA TAT AT-3′			T = 250 bp
***IL8***	P3: 5′-TAG AGA ACT TAT GCA CCC TCA-3′			
**Promoter**				
**Arg279Gln G>A**	P1: CTCCTCGCCCCAGGACTCT	-	-	TaqMan assay
**(rs17576)**	ACACCC[A/G]GGACGGCAATGC			
***MMP9***	TGATGGGAAACCC			
**Exon 6**				

* The primers for these SNPs have been designed using Primer 3 software.

One SNP, *MMP9* Gly279Arg was genotyped using the TaqMan allelic discrimination Assay, using the Applied Biosystems 7300 Real-Time PCR System, USA. Two reporter dyes VIC and FAM were used to label the Allele 1 and 2 probes and a 5′ Nuclease Assay was carried out. Fluorescence was then measured followed by automatic allele calling.

### Biochemical Investigations

Serum creatinine, blood urea, triglycerides, HDL, total cholesterol, and blood glucose levels were measured using their respective kits; high sensitive C Reactive Protein (hs-CRP) was assayed using Enzyme Linked Immuno-Sorbent Assay (ELISA) (Calbiotech Inc., USA) as per instructions of the manufacturer.

### Statistical analysis

The statistical tests were performed, using the Statistical package for Social Sciences (SPSS Inc., Chicago, IL, USA) version 13.0. Discrete and continuous variables were compared between various groups using Pearson's χ^2^ test and unpaired t-test as appropriate. Parameters showing skewed distribution (diastolic and systolic blood pressure, and serum creatinine) were compared using Mann-Whitney U test. Pearson's χ^2^ test (3×2 contingency table) was used to assess association of SNPs with renal disease between the diabetic nephropathy group (cases) and diabetes without nephropathy group (controls). Significant allelic and genotypic associations calculated by Pearson's χ^2^ test were used for evaluating odds ratio (O.R.) and 95% confidence intervals (CI) as well. The genotypes at their respective loci were coded as recessive (e.g., *TGFB1* 869TT vs. 869TC+CC) and as dominant models (e.g., *TGFB1* 869CC vs. TC+TT). The seven polymorphisms were then assessed for relation by univariate logistic regression analysis and further the variables showing a statistically significant association were made to undergo multivariate regression analysis. Power of the sample size (subjects) was calculated using the Power for Association with Errors (PAWE) software (http://linkage.rockefeller.edu/pawe) [42], [43]. Hardy Weinberg Equilibrium was calculated as performed previously [44]. We evaluated the gene–gene interactions (intra and inter) among the large number of loci in our study using the multiple dimensionality reduction (MDR) method (http://www.epistasis.org/open-source-mdrproject.html).
